# Ovarian Follicular Dynamics and Its Functional Significance in Relation with Follicle Deviation, Vaginal Cytology, and Hormone Profiles in Llamas (*Lama glama*)

**DOI:** 10.3390/ani12233299

**Published:** 2022-11-25

**Authors:** Uri H. Perez-Guerra, Yesenia M. Quispe, Henry I. Gonzáles, Natalio Luque, Domingo A. Ruelas, María I. Carretero, Miguel A. Gutiérrez-Reinoso, Manuel G. Pérez-Durand, Manuel García-Herreros

**Affiliations:** 1 Facultad de Medicina Veterinaria y Zootecnia, Universidad Nacional del Altiplano, Puno 21001, Peru; 2 Facultad de Ciencias Veterinarias, Instituto de Investigación y Tecnología en Reproducción Animal, Universidad de Buenos Aires, Consejo Nacional de Investigaciones Científicas y Técnicas, Buenos Aires C1427CWO, Argentina; 3 Facultad de Ciencias Agropecuarias y Recursos Naturales, Carrera de Medicina Veterinaria, Universidad Técnica de Cotopaxi (UTC), Latacunga 050150, Ecuador; 4 Instituto Nacional de Investigação Agrária e Veterinária (INIAV), 2005-048 Santarém, Portugal

**Keywords:** follicular dynamics, follicle deviation, vaginal cytology, endocrine profiles, llama

## Abstract

**Simple Summary:**

The main differences regarding the follicular dynamics in South American camelids are the distinct phases and their respective durations compared to those in other mammals. There are no reports on the study of vaginal cytology related to sex hormone levels during the specific phases of the follicular wave, such as the follicular recruitment, or even during the follicular deviation in South American camelids. This research was designed to study the follicular dynamics during the follicular deviation process related to vaginal cytological characteristics and endocrine profiles in llamas (*Lama glama*), being a model for the study of other camelid species.

**Abstract:**

The reproductive physiology in camelid species has its particularities. The present study aimed to characterize the ovarian follicular dynamics and its functional significance in relation to follicular deviation, vaginal cytological characteristics, and sexual hormone profiles in llamas as the first report in South American camelids. Non-pregnant, multiparous llamas (*Lama glama*; n = 10; age: 48–72 mo.; BCS: 2.5–3.0) were enrolled in the study. The ultrasonographic assessment was carried out transvaginally and follicular ablation was performed (day 0) when follicles were larger than 7 mm. The follicle number and diameter were scored daily throughout the process for a proper evaluation of the deviated follicles and to monitor the presence of new follicle pools (1.5 to 2.5 mm diameter). Vaginal cytological evaluation (parabasal, intermediate, and superficial cells) was performed every other day until day 6. Endocrine profiles (17β estradiol, anti-Mullerian hormone, testosterone, and progesterone) during pre- and post-follicular deviation were determined by using the ELISA assay. Differential follicular dynamics both in the presence of a single dominant follicle (DF) and in codominance during the follicular deviation process were detected in llamas (*p* < 0.05). The percentage of superficial cells was the most related to the follicular wave phase. However, the percentage of parabasal, intermediate, and superficial cells was not related to the phases of follicular growth, dominance, and regression (*p* > 0.05). Differential patterns among the different hormone concentration levels regarding the 17β estradiol, anti-Mullerian hormone, progesterone, and testosterone during follicular deviation were observed, with the latter being significantly different along the deviation process (*p* < 0.05). In conclusion, the use of vaginal cytology assessment would not be sufficient to determine the follicular phases in llamas. Therefore, complementary analyses, such as ultrasonography and endocrine assessment, are strongly recommended to determine follicular dynamics during the follicular deviation.

## 1. Introduction

The reproductive physiology in South American camelids has its distinctiveness, unlike other domestic species, with follicular dynamics in successive and superimposed waves and induced ovulation as the main particularities [1,2,3]. The main characteristics of the follicular dynamics in South American camelids are the different phases and their respective durations. The length of the growth phase varies between three and nine days, the dominance phase between 2 and 8 days, and finally the regression phase between three and eight days in both alpacas and llamas [2,4]. The average growth rates have been described as 0.43 mm/day in alpacas and 0.5–0.9 mm/day in llamas that have allowed a general characterization of the ovarian activity in South American camelids, taking into account that there are more physiological phenomena within the follicular dynamics related to recruitment, selection, deviation, and follicular regression [3,4,5]. In monoovular species such as camelids, only one of many follicles becomes dominant. This process is known as selection and is characterized by the acquisition of a higher growth rate compared to the other follicles (subordinate follicles), the so-called follicular deviation [6,7]. This process starts with the average recruitment of 7–11 follicles per cohort with diameters of 4 mm (cattle) and 6 mm (mares), reaching follicular deviation follicular diameters of 8.5–7.7 mm in cattle and 22.5–19 mm in mares [8]. Several studies in camels reported the recruited follicle diameters between 4.4 and 3.5 mm and deviated follicles between 7.43 and 7.75 mm in the presence of a single dominant follicle (FD) and co-dominance, respectively, being important results for the knowledge of the female reproductive physiology [6,9]. However, there are no reports on the follicular deviation in South American camelids that provide important input for the application of reproductive biotechnologies. In recent years, the development of reproductive biotechnologies has required greater knowledge of reproductive physiology with the aid of techniques, such as hormone analysis and mainly ultrasonography [5,10,11]. Efficient reproductive management using artificial insemination (AI) and embryo transfer (ET) requires a comprehensive knowledge of follicular dynamics and other factors affecting follicular dynamics [11,12]. The monitoring of follicular deviation allows explaining some particularities related to ET in camelids because sometimes more than one embryo at single ovulation has been reported, together with the response to superovulation (SOV) process, with the assessment of the time taken for follicular deviation and the number of recruited follicles to predict the response to the SOV protocols [13,14,15].

Vaginal cytology has been used in different species to determine the cause of abnormal vaginal discharges, reproductive tract inflammations, and tissue masses associated with problems in the ovary, uterus, vagina, and vulva [16]. In addition, it has also been used primarily to determine the exact timing of artificial insemination or natural mating, as well as to characterize the estrous cycle phases [16,17,18]. There are few reports on vaginal cytology in South American camelids, which have rarely been used to characterize and differentiate pregnant and non-pregnant females during the follicular and luteal phases [19]. In general, vaginal cytology is a non-invasive technique that could be used to facilitate reproductive management allowing to approximate the cell types present during the phases of the follicular wave generating basic information about the changes that may exist in parabasal, intermediate, and superficial cells during the follicular wave [20]. However, due to the lack of information in llamas on this subject, it is necessary to characterize the vaginal cytology concerning the phases of follicular growth, dominance, and regression.

Several studies on the determination of estrogens and progesterone concentration during the follicular wave have been carried out in camelids, observing a direct relationship between estrogen levels and follicle growth while the progesterone concentration was low during the follicular wave [21,22,23]. Moreover, other studies showed that follicular activity would be controlled through the application of exogenous progesterone, finding a decrease in the follicular wave duration and the follicle size [15,24,25]. In addition, there are studies on anti-Mullerian hormone (AMH) indicating that follicles that do not become dominant during the follicular wave continue to secrete AMH [26]. There is a lack of knowledge regarding the determination of hormone levels during specific processes related with the follicular wave, such as the follicular deviation in South American camelids. Thus, the main objective of the present study was to characterize the ovarian follicular dynamics in llamas by studying the follicular deviation related with vaginal cytological characteristics and sexual hormone profiles.

## 2. Materials and Methods

### 2.1. Ethical Statement

This study was conducted according to the guidelines of the Declaration of Helsinki and following the Code of Ethics for animal experiments as reflected in the ARRIVE guidelines available at http://www.nc3rs.org.uk/ARRIVEchecklist (accessed on 7 September 2022). The study was approved by the Bioethics Committee for the use of experimental animals at the Universidad Nacional del Altiplano—Puno—Perú (Approval Date: 1 January 2019, Code Number: DE-000399-2019).

### 2.2. Experimental Animals

This study was conducted between May and August (2020) in the Faculty of Veterinary Medicine and Animal Husbandry of the National University of the Altiplano (Puno, Perú) located at ~3812 m.a.s.l. Non-pregnant, multiparous llamas (*Lama glama*; n = 10; Age: 48–72 mo.; BCS: 2.5–3.0 according to the scale recommended by [27]) were enrolled in the study. All animals were located in “La Raya” Experimental Centre and fed natural pasture, water ad libitum, and supplemented with oat hay daily.

### 2.3. Preparation Protocol and Ultrasonographic Procedure

**Figure 1:**
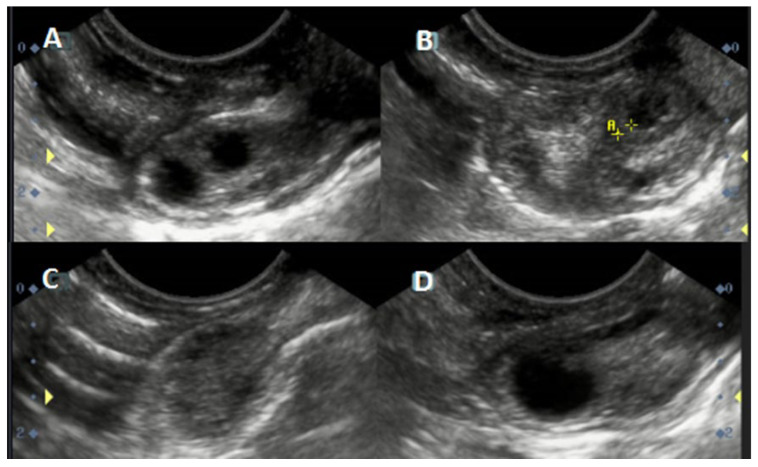
Ultrasonographic images (single dominant follicle vs. codominance). Llama 1 (codominance example within the same animal): (**A**) presence of follicular codominance in the right ovary and (**B**) presence of follicles (<2 mm, see yellow A**) in the left ovary. Llama 2 (single dominant follicle example within the same animal): (**C**) presence of follicles (<2 mm) in the right ovary and (**D**) presence of a single dominant follicle in the left ovary.

Before the ultrasonographic procedure, the llamas were immobilized. First of all, the perineal area and vulva of each animal were sanitized. Then, the ultrasonographic assessment was carried out transvaginally by using a SonoStar SS8 device (SonoStar Technologies, Guangzhou, China) equipped with an endocavitary linear microconvex transducer (V6S9 multifrequency: transvaginal examination; 6.5 MHz in 2B mode) to observe one side for each ovary (see Figure 1) using the freeze option to check images and if necessary for recording them for further evaluation [28]. Different animals belonging to the single dominant follicle (DF; F1: one single dominant follicle; n = 5) and codominance (F1 and F2: two dominant follicles; n = 5) were identified to carry out the present study. Representative images regarding different follicular dynamics are shown in Figure 1.

### 2.4. Follicular Ablation and Recruitment

A previous assessment of the general ovarian functional characteristics was performed before the beginning of the evaluations by observing one follicular wave. Thus, as recommended by previous studies when follicles were larger than 7 mm the follicular ablation was performed (day 0) [29,30,31]. This was performed with slight modifications related to the vacuum pump pressure (110 mm Hg). The recruitment was evaluated daily to check the presence of a new pool of follicles with diameters between 1.5 and 2.5 mm recording the number of follicles and the time elapsed, identifying every single follicle (F1 or/and F2: single dominant or codominant follicles; and F3 or/and F4: secondary non-dominant follicles) as recommended by Goodman et al. [32].

### 2.5. Characterization of Follicular Deviation

The assessments were performed daily from day 0 (the day of follicular ablation) every morning for each animal. First of all, the follicle identification was carried out as follows: (a) a single dominant follicle (DF) was classified as F1 (future deviated follicle) and (b) in case of codominance (two dominant follicles) was classified as F1 and F2 [9,32]. The follicle identification was performed as rounded and anechogenic structures. The diameter measurement was performed inside the follicular walls by carrying out two evaluations and calculating the average as the final follicle diameter [11,33]. The initial follicle identification was maintained throughout the process for a proper evaluation of the deviated follicles. The data matrix was subsequently systematized in spreadsheets for further statistical analyses.

### 2.6. Vaginal Cytology Evaluation

**Figure 2:**
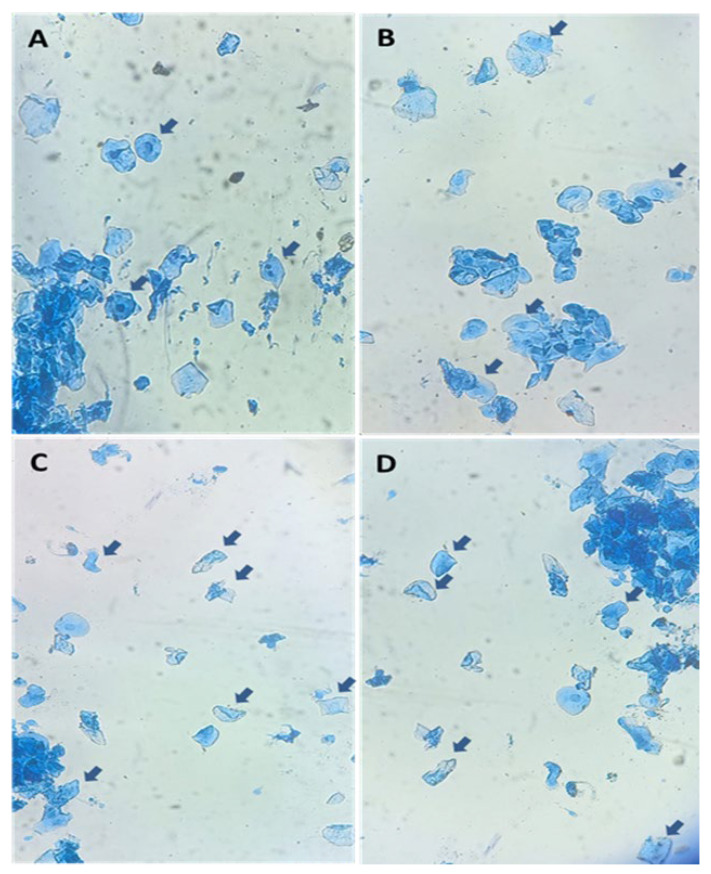
Representative cytological images (Diff–Quick staining) obtained from the dorsal area of the middle third of the vagina by using swabs in llamas (*Lama glama*): (**A**) presence of parabasal cells (arrows), (**B**) presence of intermediate cells (arrows), (**C**,**D**) presence of superficial/cornified cells (arrows).

Different cell types obtained from cytological samples are represented in Figure 2. Sampling was carried out under hygienic conditions by using gloves, and previously, cleaning the vulvar lips of the animals. Then, the sterile swab was introduced 7–10 cm towards the dorsal area of the middle third of the vagina avoiding contact with the vestibule and vulvar lips as recommended by England et al. [17,34]. Once the sample was taken, it was placed on a slide by circular movements and immediately immersed in Diff–Quick fixative solution for 1 min. following the manufacturer’s instructions (Giemsa stain, modified, Sigma-Aldrich, St. Louis, MO, USA). Then, the samples were immersed for another 1 min in Diff–Quick staining solution and the same process was repeated for the second Diff-Quick staining. Finally, the slide was gently washed with distilled water and dried until evaluation. The cells obtained were evaluated by using a phase contrast microscope at 400 X (Leica, Leica Microsystems CMS Gmbh, Wetzlar, Germany; Figure 2). The cells were assessed for morphology and size using software (Leica LAS EZ^®^ version 3.4, Wetzlar, Germany). Theoretical definitions of exfoliative cytology were as follows; (a) small circular cells (little cytoplasm) were defined as parabasal cells, (b) intermediate cells were defined as larger, irregular nucleated cells (squamous), and finally, (c) larger cells were classified as superficial cells, being anucleated in the later stages (or small reminiscent nucleus/pyknotic). The surface cells when they start to enlarge are called cornified cells [17,35].

### 2.7. Blood Sampling and Endocrine Profile Assessment by Enzyme-Linked Immunosorbent (ELISA) Assay

Blood samples were obtained daily from all individuals by jugular vein puncture (days 1, 2, 3, 4, 5, and 6 post-follicular ablation) by using heparin-containing tubes which were immediately centrifuged. Blood plasma was stored at −20 °C for further evaluation. The quantification of different plasma hormone concentrations (17β estradiol, anti-Mullerian hormone, progesterone, and testosterone) was performed by using the enzyme-linked immunosorbent (ELISA) assay. Previously, the ELISA protocols were validated for llama blood plasma samples. The blood samples collected before, during, and after the ovulation; day 1 (1st sample), day 2, day 3, day 4, day 5, and day 6 (last sample) were used for different hormone levels determination. The 17β estradiol concentration was determined by using a commercial ELISA kit (Estradiol ELISA, 17β estradiol antigenic, DiaMetra, Perúgia, Italy) following the manufacturer’s instructions. The sensitivity of the test was stated at 8.68 pg/mL and the precision (intra-assay variability) was set as ≤9.0%, and the inter-assay variability ≤ 10.0%. The anti-Mullerian hormone was determined by using a commercial ELISA kit (Anti-Mullerian Hormone [AMH] BioAssayTM ELISA Kit, Hamburg, Germany) following the manufacturer’s instructions. The sensitivity of the test was stated at 53.3 pg/mL and the precision (intra-assay variability) was set as <10% and the inter-assay was <12%. The progesterone levels were determined using a commercial ELISA Kit (Progesterone ELISA kit Enzo, Farmingdale, NY, USA) following the manual instructions. The sensitivity was stated at 8.57 pg/mL and the precision was 5.4% and 8.3% intra-assay and inter-assay respectively. Finally, the testosterone level was determined using a commercial Testosterone ELISA Kit (Testosterone ELISA Kit, Abcam, MA, USA) following the instructions. The sensitivity was stated at 0.07 ng/mL and the precision intra-assay was ≤5.8% and the inter-assay was ≤10.5%. All evaluations were determined using the same ELISA plate reader (Organon Teknica, Microwell System, model Reader 230S, Portland, OR, USA). Plasma concentrations of the different hormones were determined by using a standard ELISA protocol. Briefly, 100 µL/well were added for each hormone standard curve (312, 156, 78, 39, 19, 9.7, 4.9, 2.4, 1.2, 0.6 ng/mL in blood serum) and samples were incubated for 2 h at 37 °C to be analyzed (duplicate). After that, the plates were washed three times by using Phosphate Buffered Saline (PBS)/Polysorbate (Tween 0.05%, Sigma-Aldrich, St. Louis, MO, USA). Then, 100 µL of anti-hormone chicken serum antibody (1% in skim milk) were added in PBS/Tween 0.05% at 1:40,000 (*v*/*v*) dilution and subsequently incubated for 1 h at 37 °C. After that, the plates were washed four times by using PBS/Tween 0.05%. Then, 100 µL/well of anti-IgY antibody (1% in skim milk) were added in PBS/Tween 0.05% at 1/20,000 (*v*/*v*) dilution and subsequently incubated for 1 h at 37 °C. Once again, the plates were washed four times by using PBS/Tween 0.05%. Finally, 100 µL/well of o-Phenylenediamine dihydrochloride (OPD; Sigma-Aldrich, St. Louis, MO, USA) solution (25 mL substrate buffer + 10 mg OPD + 10 µL H2O2) was added and incubated in the dark for 10 min. The incubation was stopped by adding 2.5 M H2SO4 (50 µL/well). The ELISA plates were read at a wavelength of 492 nm.

### 2.8. Statistical Analysis

The data base was analyzed using descriptive statistics to facilitate the data interpretation by comparing both single dominant follicle (DF) and codominance. Data were subjected to normality (Shapiro–Wilk) and homoscedasticity (Levene) tests resulting in not being statistically significant. In the presence of a single DF, both follicular diameters and pre- and post-deviation growth rates were subjected to a paired *t*-test (comparing F1 vs. F2), while in the case of codominance data were subjected to a one-factor ANOVA (comparing F1 vs. F2 vs. F3 and F4). The hormone concentrations were only analyzed from the day of follicular ablation (day 0) to follicular deviation. The Chi-square test was used to determine the dependence between cell types (parabasal, intermediate, and superficial cells) and follicular dynamics’ different stages. Statistical analyses were performed using R statistical software version 4.0.2 [36] with the ‘Rcmdr’ package. The significance level was set at *p* ≤ 0.05.

## 3. Results

### 3.1. Follicular Wave: General Characteristics

The ultrasonographic evaluation was carried out to determine the functionality of both ovaries being 53.85% and 46.15% for the right and left ovary, respectively. Regarding the evaluation of the presence of codominance (deviation of two follicles in the same follicular wave: F1 and F2) versus the growth of a single (DF) follicle (F1), 30.77% and 69.23% was observed for codominance and single cases, respectively (*p* < 0.05). The diameter of recruited follicles (day 1 post follicular ablation) was 2.21 ± 0.43 and 2.16 ± 0.61 mm for the right and left ovary, while the number of follicles observed was 6.33 ± 2.06 and 6.78 ± 1.56 in the right and left ovary, respectively (*p* > 0.05).

### 3.2. Follicular Diameter (Single DF vs. Codominance): General Characteristics

Table 1 shows the follicular diameter during the monitoring process (days 1–6) for both single DF and codominance cases. No statistical differences were observed when follicular diameter was compared between single DF and codominance cases within the same day (*p* > 0.05). Thus, a similar pattern of follicular growth (deviation) was observed when a single DF and codominance were present (*p* = 0.4582). However, statistical differences were observed when follicular diameter was compared with the evaluation day (*p* < 0.05). There was a substantial increase in follicular diameter when day 1 and day 2 were compared to the remaining days in both single DF and codominance cases (*p* = 0.00237).

### 3.3. Follicular Diameter (Single DF vs. Codominance) during Pre- and Post-Follicular Deviation

Table 2 shows the follicular diameter in pre- and post-follicular deviation for both single DF and codominance cases. The similar diameter was observed before deviation in both cases being 2.21 ± 0.89 and 2.24 ± 0.89 mm for F1 and F2, respectively, while in codominance cases 2.65 ± 0.93, 2.25 ± 0.67, 2.37 ± 0.66, and 2.32 ± 0.57 mm were observed for F1, F2, F3, and F4, respectively. The post-follicular deviation diameter values showed significant differences in the case of single DF 6.01 ± 0.73 mm for F1 and 2.05 ± 0.52 mm for F2 (subordinate follicle) while for codominance cases it was 6.65 ± 2.61 and 5.59 ± 2.17 mm for F1 and F2, respectively (dominant follicles: codominance) and 2.93 ± 1.02 and 2.84 ± 0.91 mm for F3 and F4, respectively (subordinate follicles).

### 3.4. Follicular Growth Profiles (Single DF vs. Codominance) during Pre- and Post-Follicular Deviation

Table 3 shows the follicular deviation in the presence of a single DF vs codominance. On day 4, a significant increase in F1 was observed compared to the subordinate follicles (F2, F3, and F4), noting larger follicular sizes due to a slight superiority of their growth rate (0. 656 mm/day) compared to the codominance follicular deviation (0.58 and 0.64 mm/day for F1 and F2, respectively). Regarding single DF, the regression rate of subordinate follicles (F2) was −0.01 mm/day compared to codominance where the regression rate of subordinate follicles (F3 and F4) was −0.22 and −0.24 mm/day, respectively. Finally, the follicular deviation diameter on day 4 post follicular ablation in the presence of a single DF was 4.01 mm. Moreover, the ultrasonographic chronology after the follicular ablation (day 0: follicular wave synchronization) determined the follicular deviation when there is codominance. F1 diameter differentiation started on day 4. However, the F2 diameter differentiation began on day 5. The follicular differentiation for both F1 and F2 continues increasing the deviation values compared to the subordinate follicles. Moreover, F2 maintained a smaller diameter compared to F1 during the whole stage of follicular deviation. The follicular deviation diameter in the presence of codominance was 3.88 and 3.47 mm for F1 and F2, respectively, on day 4 post-follicular ablation.

### 3.5. Vaginal Cytological Profiles and Follicular Dynamics

The relationship between the cell percentage obtained by vaginal cytology to the main phases of follicular dynamics (single DF vs. codominance) obtained by transrectal ultrasonography in llamas is shown in Table 4. The percentage of parabasal cells was 20.12%, 20.33%, and 18.02% in single DF animals during the growth, dominance, and follicular regression phase, respectively, while the percentage of intermediate cells during the same phases was 42.06%, 41.99%, and 44.89%, respectively. No differences were observed regarding the percentage of parabasal and intermediate cells along the different follicular phases (*p* > 0.05). Regarding the superficial cells, the percentage was 41.25% for the growth phase, 43.00% for the dominance phase, and 39.11% during the follicular regression phase. The type of cells more related to the follicular wave phase in llamas were the superficial cells, while the parabasal and intermediate cells were indifferent to the phases of follicular development. No differences were observed regarding the percentage of superficial cells along the different follicular phases (*p* > 0.05).

The relationship between the cell percentage (%) obtained by vaginal cytology with the main phases of follicular dynamics (codominance) obtained by transrectal ultrasonography in llamas is shown in Table 4. Similarly, in single DF animals, no differences were observed regarding the percentage of parabasal and intermediate cells along the different follicular phases (*p* > 0.05). Moreover, no differences were observed regarding the percentage of superficial cells along the different follicular phases (*p* > 0.05).

### 3.6. Chronological Hormone Levels during the Follicular Deviation (Single DF vs. Codominance)

Table 5 shows hormone concentrations regarding the follicular deviation in the presence of a single DF vs. codominance during the evaluation process.

No significant differences were observed between single DF or codominance when anti-Mullerian hormone, 17β estradiol, and progesterone concentrations were compared (*p* > 0.05). However, significant differences were observed (with a tendency to increase) in testosterone concentration levels from day 3 post-follicular ablation onwards in the case of both single DF and codominance (*p* = 0.043).

### 3.7. Hormone Profiles during the Follicular Deviation Process

**Figure 3:**
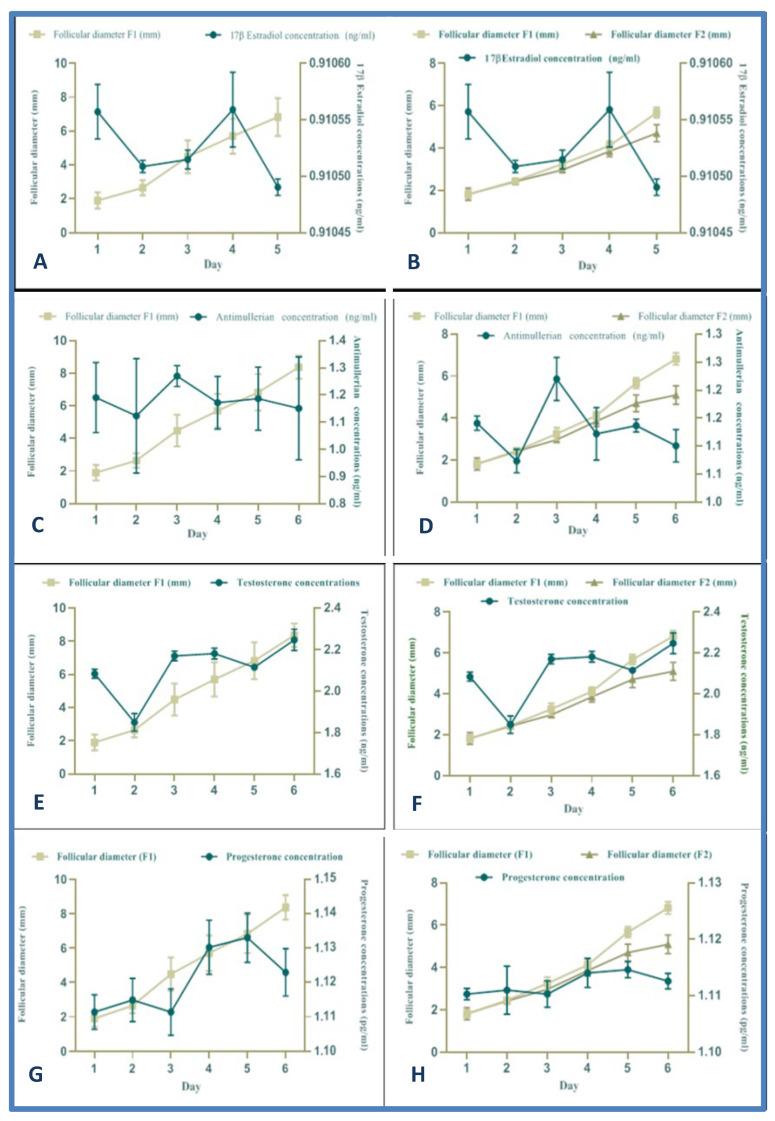
Follicle diameter vs. 17β estradiol (**A**,**B**), anti-Mullerian (**C**,**D**), testosterone (**E**,**F**), and progesterone (**G**,**H**) concentration in relation with the duration of the follicular deviation (single DF vs. codominance) in llamas (*Lama glama*). Single DF (left; (**A**,**C**,**E**,**G**)) vs. codominance (right; (**B**,**D**,**F**,**H**)). Mean (± S.E.M.) follicular diameter (mm) and serum hormone concentration (ng/mL) from day 1 to day 6 in llamas.

Figure 3A,B shows the relationship between follicular diameter and 17 β estradiol concentration. The diameter differentiation of the deviated follicles (F1: single DF vs. F1 and F2: codominance) was observed on day 4 post-follicular ablation. The 17β estradiol concentration increased at the beginning (before follicular 17β estradiol ablation) due to the presence of the DF of the previous follicular wave. Subsequently, a similar behavior relating to the average follicular diameter evaluated for both cases of follicular deviation was observed (single DF vs. codominance), day 4 being the beginning of the future DFs differentiation, increasing their diameters as opposed to the decrease in the diameter of the subordinate follicles (follicular deviation in llamas). The concentration of anti-Mullerian hormone in the presence of a single DF and codominance in relation with follicular diameter is shown in Figure 3C,D. A relationship between follicular diameter and the anti-Mullerian hormone plasma concentration was observed from day 2 to day 6 in the presence of a single DF (*p* < 0.05). Regarding codominance, a parallel relationship was observed from day 2 to day 6 between the anti-Mullerian hormone levels and the process of follicular deviation together with the increase in the follicular diameter of the future DFs (*p* < 0.05). Figure 3E,F shows the relationship between follicular diameter and plasma testosterone concentration in the presence of a single DF and codominance, respectively. In both cases, regardless of the type (single DF vs. codominance) higher testosterone hormone levels were observed on day 4 compared to the other time points, which coincides with the timing of follicular deviation (*p* < 0.05). On the other hand, in both cases (single DF and codominance), there was an immediate decrease in testosterone hormone concentration on day 5 (*p* < 0.05). Finally, regarding progesterone levels, Figure 3G,H shows the relationship between follicular diameter and plasma progesterone concentration in the presence of a single DF and codominance, respectively. Similar behavior to testosterone was observed. However, the main difference compared to testosterone levels was that the progesterone concentration increase was maintained until day 6 of follicular development (*p* < 0.05).

## 4. Discussion

This is the first report of follicular deviation in llamas. The follicular deviation is defined as a change in the growth rate of the future dominant follicles (F1: single DF; F1 and F2: codominance) as opposed to subordinate follicles [6]. The alternation regarding ovarian function in camelids has been previously described in several studies [3,4,5,22,37]. The presence of single dominance (a single DF, F1) and codominance (two follicles, F1 and F2) is a feature reported in camelids and cattle [5,9,38]. This phenomenon consists of the growth of two follicles in the same follicular wave observing a direct relationship with the increase of 17β estradiol concentrations from day 3 and 4 post-follicular ablation. Codominance is a phenomenon widely studied in cattle where there is a slight increase in gonadotropins (FSH and LH) before follicular deviation [38,39], something that could probably be happening in South American camelids. However, the behavior of gonadotrophins before and after follicular deviation in camelids has not yet been studied.

In the present study, differentiated rates of future DFs of 0.656 mm/day for a single DF and 0.58 mm/day for F1 and 0.64 mm/day for F2 in the case of codominance have been observed, which are similar growth rates to those reported by other authors [5,9]. In addition, during the growth phase of the follicular wave, similar regression rates have been described for subordinate follicles in cattle and camelids [5,6,9]. The onset of follicular deviation in llamas starts on day 4 post-follicular ablation both in the presence of a single DF with a diameter of 4.01 mm and in codominance with diameters for F1 of 3.88 mm and F2 of 3.47 mm, respectively. The onset of follicular deviation is similar to that reported in camels as the species most comparable to llamas [9] as well as in cattle [40,41,42]. In the present study, the follicular dimensions in llamas are objectively determined for the first time on day 5 post-follicular ablation, being 6.01 mm for a single DF, and 6.65 and 5.59 mm for F1 and F2, respectively, in the case of codominance.

The percentage of parabasal cells obtained in the present study is similar compared to those reported for camels and non-pregnant alpacas during the follicular phase. However, differences were observed when compared to intermediate and superficial cells, probably because the exfoliative cytology samples were taken at different times of follicular dynamics, such as growth, dominance, and regression phases [43,44]. The results obtained show that there is no dependence or relationship between cell type and the follicular phase. This phenomenon seems to be specific in camelid species as they generally do not have a progesterone-dominant luteal phase (presence of corpus luteum) except during pregnancy [45]. Another reason for this could be due to the effects of stimulation with exogenous hormones, such as GnRH or LH, that produce ovulation with the corpus luteum reaching a half-life of approximately 13 days, when the maximum progesterone peak reaches 10 ng/mL on day 9–10 post-hormonal stimulation [25]. However, camelids are characterized by overlapping waves with ovarian alternation that prevents a marked pattern concerning the percentages of cells assessed by vaginal cytology as occurs in other species [3,46,47].

The differentiation observed regarding the percentage of surface cells in other species during the follicular growth, dominance, and regression phases is probably due to effects derived from the level of estrogens during the estrus together with the low androstenedione and estradiol concentration that help to stimulate the cell proliferation [19,20]. Similarly, an increase in the percentage of surface cells has been observed when estrogen concentration increases during follicular dominance in llamas, with a relationship between the phases of the follicular wave and estrogen levels [3,21,22]. This phenomenon has also been observed in felines where the percentage of surface cells increases slightly during the follicular phase when the transition from estrus to post-estrus occurs [48]. This exfoliation process is an indicator of cell necrosis that occurs during the period of transition from round cells (basal and parabasal) to irregular cells, whose nuclei progressively decrease in size [49,50]. The dimensions observed in parabasal and intermediate cells are similar to those reported in other species and even in other camelid species such as the alpaca [10]. However, the size of the superficial cells in the present study is smaller compared to that reported in other studies in other species [51]. The other cell characteristics evaluated are similar to those reported by several authors who mention that parabasal cells are characterized by their round or nearly round shape and large nucleus, with the intermediate cells being two times larger than parabasal cells [16]. The main characteristic of intermediate cells is that they have a large size, polygonal shape, and small nucleus. On the other hand, superficial cells are larger, polygonal in shape, and have pyknotic nuclei, although anucleate cells with angular and irregular borders have also been observed [20,51]. However, in the present study, the use of vaginal cytology assessment was not sufficient to determine the follicular phases in llamas. Therefore, complementary analyses, such as ultrasonography and hormone profile determination, are strongly recommended.

Regarding the levels of 17β estradiol during the follicular deviation process, a pattern compatible with follicular growth has been observed during the follicular recruitment phase (days 1, 2, and 3) increasing together with the levels of 17β estradiol on day 4 (post-follicular ablation). However, this pattern of hormonal behavior and follicular dimension obtained between 17β estradiol and follicular diameter during the growth, dominance, and regression phase is similar to those reported in alpacas, guanacos, and vicuñas during the follicular wave [5,21,22,37,45]. Moreover, in the present study, the concentration of testosterone increases in a similar way to 17β estradiol. Several studies have reported that androgens cause the accumulation of FSH in the pituitary gland, which is related to the increase of 17β estradiol [5]. In addition, the androgens are precursors for the production of 17β estradiol by granulosa cells (mainly androstenedione and testosterone) at the level of the inner theca cells. This process occurs due to the selective stimulation of aromatase [52]. However, other studies indicate that there is no relationship between the process of follicular deviation and the increase in testosterone concentration [11].

In the present study, the progesterone concentration increases along with the production of 17β estradiol, and this is related to follicular deviation during the camelid estrous cycle [37]. On the other hand, the progressive increase in anti-Mullerian hormone (AMH) concentration along with the processes of follicular recruitment and follicular deviation may be because this hormone is characteristically synthesized in the granulosa cells of the growing follicles, mainly the pre-antral and the smaller antral follicles [53]. AMH concentrations show a direct relationship with follicular deviation (increase in the diameter of the deviant follicle), probably because the follicle has granulosa cells, which are responsible for the synthesis of AMH. Other authors have carried out other related studies. However, they have additionally reported that in women the expression of AMH in larger antral follicles disappears, which would indicate that AMH is only expressed in follicles already recruited as they have not yet been selected to be the future dominant follicles [11,54]. In the present study, the progesterone concentration increases along with the production of 17β estradiol, and this is related to follicular deviation. This is probably because there is an increase in progesterone levels mainly synthesized by the granulosa cells due to FSH and LH stimulation. For this reason, progesterone could be a substrate for continued estradiol secretion by the future dominant follicle [37,52].

## 5. Conclusions

The present study objectively shows for the first time the values corresponding to the follicular dynamics in llamas (*Lama glama*) both in the presence of a single DF and in codominance during the follicular deviation process. The main biological significance of the present study is related to the discovery of unique particularities of the reproductive cycle which are particularly different in South American camelids (single DF vs. codominance) compared to other mammalian species. Beyond the biological information found in the present research, this knowledge can be used to improve assisted reproductive techniques (ARTs) in South American camelids, such as induced superovulation and in vivo embryo production. The superficial cells’ percentage was the most related to the follicular wave phase in llamas. The percentage of parabasal, intermediate, and superficial cells was not differentially related to the phases of follicular growth, dominance, and regression. In addition, the hormonal levels corresponding to the different follicular dynamics were determined, having differential patterns among the different endocrine concentration levels regarding the 17β estradiol, anti-Mullerian hormone, progesterone, and testosterone during the follicular deviation, with the latter being significantly different along the deviation process. Finally, the use of vaginal cytology assessment per se would not be sufficient to determine the phases of follicular growth, dominance, and regression. Therefore, the use of complementary analyses, such as ultrasonography and endocrine profile assessment, is strongly recommended in llamas.

## Figures and Tables

**Table 1:** Follicle diameter in llamas (*Lama glama*): single dominant follicle vs. codominance during the monitorisation process (Day 1 to Day 6).

Day	Follicular Diameter (Single DF)	Follicular Diameter (Codominance)	
		F1	F2
1	1.90 ± 0.28 ^a^	1.82 ± 0.17 ^a^	1.82 ± 0.28 ^a^
2	2.65 ± 0.05 ^a^	2.45 ± 0.03 ^a^	2.42 ± 0.05 ^a^
3	4.49 ± 0.11 ^ab^	3.25 ± 0.28 ^ab^	2.97 ± 0.11 ^ab^
4	5.70 ± 0.25 ^b^	4.12 ± 0.2 ^ab^	3.85 ± 0.25 ^ab^
5	6.83 ± 0.40 ^b^	5.67 ± 0.25 ^b^	4.70 ± 0.40 ^ab^
6	8.37 ± 0.44 ^b^	6.82 ± 0.28 ^b^	5.10 ± 0.44 ^b^

Values are presented as Mean ± S.E.M. Different superscripts (^a,b^) within a column show statistical differences among follicle diameters (*p* ≤ 0.05). Single dominant follicle (n = 5) vs. codominance (n = 5). Follicle diameters are shown in mm.

**Table 2:** Follicle diameter in llamas (*Lama glama*): single DF vs. codominance deviation.

Follicular Structure	Single Dominant Follicle		Codominance	
	Pre-Deviation	Post-Deviation (Day 5)	Pre-Deviation	Post-Deviation (Day 5)
F1	2.21 ± 0.89	6.01 ± 0.73 ^a^	2.65 ± 0.93	6.65 ± 2.61 ^a^
F2	2.24 ± 0.89	2.05 ± 0.52 ^b^	2.25 ± 0.67	5.59 ± 2.17 ^a^
F3			2.37 ± 0.66	2.93 ± 1.02 ^b^
F4			2.32 ± 0.57	2.84 ± 0.91 ^b^
*p* value	0.9339	˂0.0001	0.635	˂0.000001

Values are presented as Mean ± S.E.M. Different superscripts (^a,b^) within a column show statistical differences among follicle diameters (*p* ≤ 0.05). Follicle diameters are shown in mm.

**Table 3:** Follicular growth rates in llamas (*Lama glama*): single DF vs. codominance deviation.

Follicular Structure	Single Dominant Follicle		Codominance	
	Pre-Deviation	Post-Deviation (Day 5)	Pre-Deviation	Post-Deviation (Day 5)
F1	0.66 ± 0.37	0.65 ± 0.31 ^a^	0.52 ± 0.42	0.58 ± 0.34 ^a^
F2	0.44 ± 0.32	−0.01 ± 0.29 ^b^	0.36 ± 0.35	0.64 ± 0.67 ^a^
F3			0.35 ± 0.35	−0.22 ± 0.45 ^b^
F4			0.24 ± 0.39	−0.24 ± 0.49 ^b^
*p* value	0.05	˂0.000001	0.286	˂0.000001

Values are presented as Mean ± S.E.M. Different superscripts (^a,b^) within a column show statistical differences among follicle growth rates (*p* ≤ 0.05). Follicle growth rates are shown in mm.

**Table 4:** Percentage of different cell types (parabasal, intermediate, and superficial) during follicular dynamics (single DF vs. codominance) obtained from vaginal cytological assessment in llamas.

Follicular Phase	Single Dominant Follicle			Codominance		
	Cell Type					
	Parabasal (%)	Intermediate (%)	Superficial (%)	Parabasal (%)	Intermediate (%)	Superficial (%)
Growth (~7 mm)	20.12 ± 1.93 ^a^	42.06 ± 6.37 ^b^	41.25 ± 3.21 ^b^	24.12 ± 2.81 ^a^	39.06 ± 8.22 ^b^	42.25 ± 5.11 ^b^
Dominance (~12 mm)	20.33 ± 1.09 ^a^	41.99 ± 7.12 ^b^	43.00 ± 4.96 ^b^	23.33 ± 2.00 ^a^	37.99 ± 9.06 ^b^	45.00 ± 8.36 ^b^
Regression (~10 mm)	18.02 ± 0.90 ^a^	44.89 ± 8.03 ^b^	39.11 ± 6.10 ^b^	23.02 ± 1.92 ^a^	38.89 ± 8.53 ^b^	40.11 ± 7.64 ^b^

Values are presented as % (Mean ± S.E.M.) within a row with different superscript letters (^a,b^) among cell types are significantly different (*p* < 0.05). No differences were observed regarding the percentage of each cell type within columns (*p* > 0.05). Follicular phases were determined by follicle diameter evaluation (diameter shown between parentheses).

**Table 5:** Hormone concentration profiles during the follicular deviation process in llamas (*Lama glama*) from Day 1 to Day 6: single dominant follicle vs. codominance deviation.

Day	Hormone Concentration							
	Testosterone (ng/mL)		Anti-Mullerian (ng/mL)		17 β Estradiol (ng/mL)		Progesterone (pg/mL)	
	Single DF	Codominance	Single DF	Codominance	Single DF	Codominance	Single DF	Codominance
1	2.080 ± 0.020 ^a^	2.078 ± 0.020 ^a^	1.210 ± 0.010	1.190 ± 0.020	0.9105 ± 0.000	0.9105 ± 0.000	1.110 ± 0.001	1.110 ± 0.001
2	1.848 ± 0.040 ^b^	1.845 ± 0.040 ^b^	1.110 ± 0.020	1.120 ± 0.010	0.9104 ± 0.000	0.9105 ± 0.000	1.119 ± 0.001	1.111 ± 0.001
3	2.156 ± 0.020 ^a^	2.183 ± 0.030 ^a^	1.260 ± 0.040	1.290 ± 0.030	0.9105 ± 0.000	0.9105 ± 0.000	1.110 ± 0.001	1.110 ± 0.001
4	2.175 ± 0.020 ^a^	2.182 ± 0.020 ^a^	1.190 ± 0.050	1.180 ± 0.030	0.9105 ± 0.000	0.9105 ± 0.000	1.113 ± 0.001	1.114 ± 0.001
5	2.115 ± 0.020 ^a^	2.117 ± 0.010 ^a^	1.185 ± 0.010	1.194 ± 0.030	0.9104 ± 0.000	0.9105 ± 0.000	1.114 ± 0.001	1.115 ± 0.001
6	2.256 ± 0.050 ^c^	2.211 ± 0.020 ^c^	1.150 ± 0.030	1.150 ± 0.010	0.9105 ± 0.000	0.9105 ± 0.000	1.112 ± 0.001	1.112 ± 0.001

Values are presented as Mean ± S.E.M. Different superscripts (^a–c^) within a column show statistical differences among endocrine concentrations within the same hormone during the follicular deviation (*single DF* vs. *codominance; p* ≤ 0.05).

## Data Availability

All data generated or analyzed during this study are included in this article.
